# Randomized phase III trial evaluating the role of weight loss in adjuvant treatment of overweight and obese women with early breast cancer (Alliance A011401): study design

**DOI:** 10.1038/s41523-017-0040-8

**Published:** 2017-09-21

**Authors:** Jennifer A. Ligibel, William T. Barry, Catherine Alfano, Dawn L. Hershman, Melinda Irwin, Marian Neuhouser, Cynthia A. Thomson, Linda Delahanty, Elizabeth Frank, Patty Spears, Electra D. Paskett, Judith Hopkins, Vanessa Bernstein, Vered Stearns, Julia White, Olwen Hahn, Clifford Hudis, Eric P. Winer, Thomas A. Wadden, Pamela J. Goodwin

**Affiliations:** 1 Dana-Farber Cancer Institute, Boston, MA USA; 2 Alliance Statistics and Data Center, Dana-Farber Cancer Institute, Boston, MA USA; 3 American Cancer Society, Washington, DC USA; 4 Columbia University Medical Center, New Yok, NY USA; 5 Yale University, New Haven, CT USA; 6 Fred Hutchinson Cancer Research Center, Seattle, WA USA; 7 University of Arizona, Tucson, AZ USA; 8 Massachusetts General Hospital Cancer Center, Boston, MA USA; 9 North Carolina State University, Raleigh, NC USA; 10 The Ohio State University Medical Center, Columbus, OH USA; 11 Novant Health Oncology Specialists, Winston-Salem, NC USA; 12 University of British Columbia, Vancouver, Victoria BC Canada; 13 Johns Hopkins School of Medicine/Sidney Kimmel Cancer Center, Baltimore, MD USA; 14 University of Chicago, Chicago, IL USA; 15 American Society of Clinical Oncology, Alexandria, VA USA; 16 University of Pennsylvania, Philadelphia, PA USA; 17 University of Toronto/Mount Sinai Hospital, Toronto, Ontario Canada

## Abstract

Excess body weight is a poor prognostic factor in women with early breast cancer, but the effect of weight loss on the risk of breast cancer recurrence and mortality in women who are overweight or obese at the time of breast cancer diagnosis has not been evaluated. The Alliance for Clinical Trials in Oncology Breast Cancer Weight Loss trial, also known as A011401, is testing the impact of a telephone-based weight loss program on invasive disease-free survival in 3136 women with a body mass index ≥27 kg/m^2^ who have recently been diagnosed with stage II-III, HER-2 negative breast cancer. Secondary outcomes of the trial include the impact of the weight loss intervention on overall survival, body weight, physical activity, dietary intakes, incidence of comorbidities, serum biomarkers and patient reported outcomes. Participants are randomized 1:1 to a 2-year, telephone-based weight loss intervention or to an education control group. The intervention is delivered through 42 telephone calls, delivered by health coaches based at the Dana-Farber Cancer Institute. Calls are supplemented by an intervention workbook, as well as a number of tools to help facilitate weight loss. Intervention goals include loss of 10% of baseline body weight, achieved through caloric restriction and increased physical activity. This large-scale study testing the impact of purposeful weight loss after cancer diagnosis on the risk of breast cancer recurrence and mortality has the potential to make weight loss programs a standard part of breast cancer treatment.

## Background and study rationale

Growing evidence suggests that obesity is associated with increased risk of recurrence and mortality in women diagnosed with early stage breast cancer.^1-3^ Chan et al. published a recent meta-analysis of 82 studies evaluating the relationship between body weight and breast cancer outcomes, encompassing 213,075 women.^1^ A 35% increase in breast cancer-related mortality and 41% increase in overall mortality was demonstrated in women who were obese at the time of breast cancer diagnosis as compared to women who were of normal weight. Analyses by menopausal status did not demonstrate significant heterogeneity between premenopausal and postmenopausal women; however, risk estimates appeared greater in premenopausal women [RR-1.75 (95% confidence interval (CI)) 1.26–2.41] as compared to post-menopausal women [RR -1.34 (95% CI 1.18–1.53)].

There are few data from observational studies describing the relationship between weight loss after breast cancer diagnosis and disease outcomes. Two randomized trials that tested the impact of dietary modification on disease-free survival in women with early-stage breast cancer shed some light on this issue. The Women’s Interventional Nutrition Study (WINS) randomized 2400 breast cancer patients to a low-fat dietary intervention or usual care control group.^4^ Intervention participants experienced on average a 2.7 kg (3.7%) weight loss over 5 years and a 24% reduction in breast cancer recurrence as compared to control participants. In contrast, the Women’s Healthy Eating and Living (WHEL) Study randomized 3088 women to a high fruit and vegetable, low-fat diet or usual care control group.^5^ Participants assigned to the intervention group did not lose weight, and there was no difference in rates of recurrence between the intervention and control groups. Although there are a number of differences between the two trials, it has been hypothesized that weight loss is one of the potential drivers of the improved outcomes seen in women randomized to the low-fat dietary intervention in the WINS trial.

### Biological mechanisms linking obesity and breast cancer

The biologic mechanisms underlying the relationship between lifestyle factors and breast cancer prognosis are not well understood. Studies have demonstrated that elevated levels of fasting insulin at the time of breast cancer diagnosis, commonly observed in individuals with obesity, are linked to an increased risk of breast cancer recurrence and death.^6-8^ Goodwin and colleagues demonstrated a two-fold increase in the risk of breast cancer recurrence and a three-fold increase in the risk of death in patients within the highest quartile of fasting insulin levels compared to the lowest.^6^ Another study demonstrated that women with high levels of c-peptide at the time of breast cancer diagnosis had a lower event-free survival compared to women with lower levels of c-peptide.^9^ In addition to insulin, other metabolic hormones, such as insulin-like growth factor-1 and the adipokines leptin and adiponectin, have been associated with poor outcomes in patients with early-stage breast cancer.^10-14^

Chronic inflammation, also associated with obesity, has also been linked to breast cancer risk and prognosis.^15^ Obesity is associated with subclinical inflammation in human breast white adipose tissue.^16^ Preclinical data demonstrate that higher levels or genetic alterations in inflammatory biomarkers such as TNFα and IL-2 are associated with increased breast cancer risk, and cohort studies have demonstrated that inflammatory biomarkers such as c-reactive protein and IL-6 are associated poor prognosis in early-stage breast cancer.^15, 17^

### Summary

Despite observational data supporting a relationship between weight and breast cancer prognosis, as well as translational work providing a biologic rationale for this relationship, there is little information regarding the impact of weight loss upon the risk of recurrence and mortality in women with early-stage breast cancer. Given that weight is a modifiable factor, further research is needed to determine if weight loss could be an effective strategy to improve prognosis in overweight and obese women with breast cancer. This manuscript describes the design of the Randomized Phase III Trial Evaluating the Role of Weight Loss in Adjuvant Treatment of Overweight and Obese Women with Early Breast Cancer (A011401). The trial, also known as the Breast Cancer Weight Loss (BWEL) study, is designed to test the impact of a telephone-based weight loss intervention on rates of breast cancer recurrence in women with early-stage breast cancer who have a body mass index (BMI) of at least 27 kg/m^2^.

## Study development

The concept for A011401 was developed through a coordinated effort of the NCI Clinical Trials Cooperative Groups (now NCTN/NCORP groups) in a process facilitated by the National Cancer Institute (NCI). A Clinical Trials Planning Meeting that included representatives from all of the U.S. National Clinical Trials Network (NCTN) groups, NIH staff focused on weight loss, patient advocates, and external experts in weight loss, exercise physiology, nutrition science, behavioral medicine and biostatistics, was convened in February 2014 by the NCI. Meeting attendees reviewed the evidence linking obesity to breast cancer outcomes, as well as data demonstrating the feasibility of implementing a large-scale weight loss trial in breast cancer patients, and concluded that there was sufficient evidence to support the development of a randomized trial testing the impact of weight loss on disease-free and overall survival (OS) in overweight and obese women with early-stage breast cancer.

Following the planning meeting, a writing group was formed that included representatives of each of the NCTN groups, patient advocates and experts in weight loss, exercise and nutrition. Through a process that took several months, a concept was drafted and feedback was collected from writing group members and the Breast Cancer Committees of the NCTN groups. A number of considerations shaped final study design. For example, competing considerations influenced eligibility criteria and sample size. Given the many known benefits of weight loss in terms of general health, there was a desire to ensure that the study would be powered to detect even a relatively modest benefit of the lifestyle intervention. At the same time, the complexity and cost of administering a lifestyle intervention to a large group of breast cancer survivors led to the need to keep the sample size as small as possible. Thus, in order to provide enough power to evaluate the primary endpoint with a manageable sample size, eligibility was restricted to patients with stage II and III disease. Additionally, in order to enroll a large enough population of women with stage II–III disease who were representative of the broad racial, ethnic, geographic and socioeconomic diversity of breast cancer patients, it was necessary to utilize a lifestyle intervention that could be delivered to patients enrolled through a large number of centers, leading to the development of a telephone-based weight loss program for the trial.

The concept developed by the writing groups was approved by the Alliance for Clinical Trials in Oncology (Alliance). The final concept was approved by NCI and finally by the Breast Cancer Steering Committee in April 2015. The study protocol was approved by the Clinical Trials Evaluation Program (CTEP) and subsequently by the NCI Adult Central Institutional Review Board. The study is being conducted in accordance with all relevant protocols.

## Objectives

The primary objective of the BWEL trial is to test the impact of a telephone-based weight loss intervention on invasive disease-free survival (IDFS), an aggregate endpoint that includes locoregional and distant breast cancer recurrence, contralateral invasive breast cancer, new primary non-breast cancer, and death from any cause.^18^

The trial also includes a number of secondary and correlative outcomes (Table 1). These include evaluating the impact of the weight loss intervention on: OS and distant disease-free survival, incidence of co-morbidities (such as cardiovascular disease and diabetes), quality of life and other patient-reported outcomes (PRO), and levels of biomarkers linked to breast cancer risk and outcomes, such as fasting insulin, adipokines and inflammatory markers.

**Table 1:** BWEL (A011401) trial at a glance

Trial details	Description
Rationale	• Observational evidence shows that women who are overweight or obese at the time of breast cancer diagnosis have a higher risk of breast cancer mortality compared to leaner women
	• The Women’s Interventional Nutrition Study (WINS) suggested that weight loss, achieved through dietary fat restriction, may be linked to a lower risk of breast cancer recurrence
	• More than two-thirds of women diagnosed with breast cancer in the US are overweight or obese
Hypothesis	Weight loss, achieved through participation in a supervised weight loss intervention, will significantly improve invasive disease free survival in overweight and obese women with early-stage breast cancer
Primary endpoint	Invasive disease free survival
Secondary endpoints	• Overall survival
	• Distant disease-free survival
	• Weight
	• Body composition
	Insulin resistance syndrome associated conditions – diabetes, hospitalization for CV disease
	• Correlative science
	Fasting metabolic and inflammatory biomarkers
	Predictive tissue markers
	• Health behaviors
	Minutes of weekly exercise
	Dietary intake
	• Patient reported outcomes
	Quality of life
	Treatment-related side effects
	Body image
	Sleep
Integrated biomarkers	Fasting insulin, leptin and high sensitivity c-reactive protein
Sample size	A target sample size of 3136 women will provide 85% power to detect a hazard ratio of 0.80
	Assumptions:
	• IDFS rate in control population of 77%
	• One-sided type I error rate of 0.025
	• 4 years accrual and 4 years of additional follow up
Patient population	• Stage II–III breast cancer diagnosed within the last 12 months
	• Her-2 negative
	• BMI ≥ 27 kg/m^2^

*US* United States, *IDFS* invasive disease free survival, *CV* cardiovascular

Secondary analyses will also evaluate the impact of the intervention on IDFS and OS in patient subsets defined by menopausal status and tumor hormone-receptor status.

## Study design

**Fig. 1:**
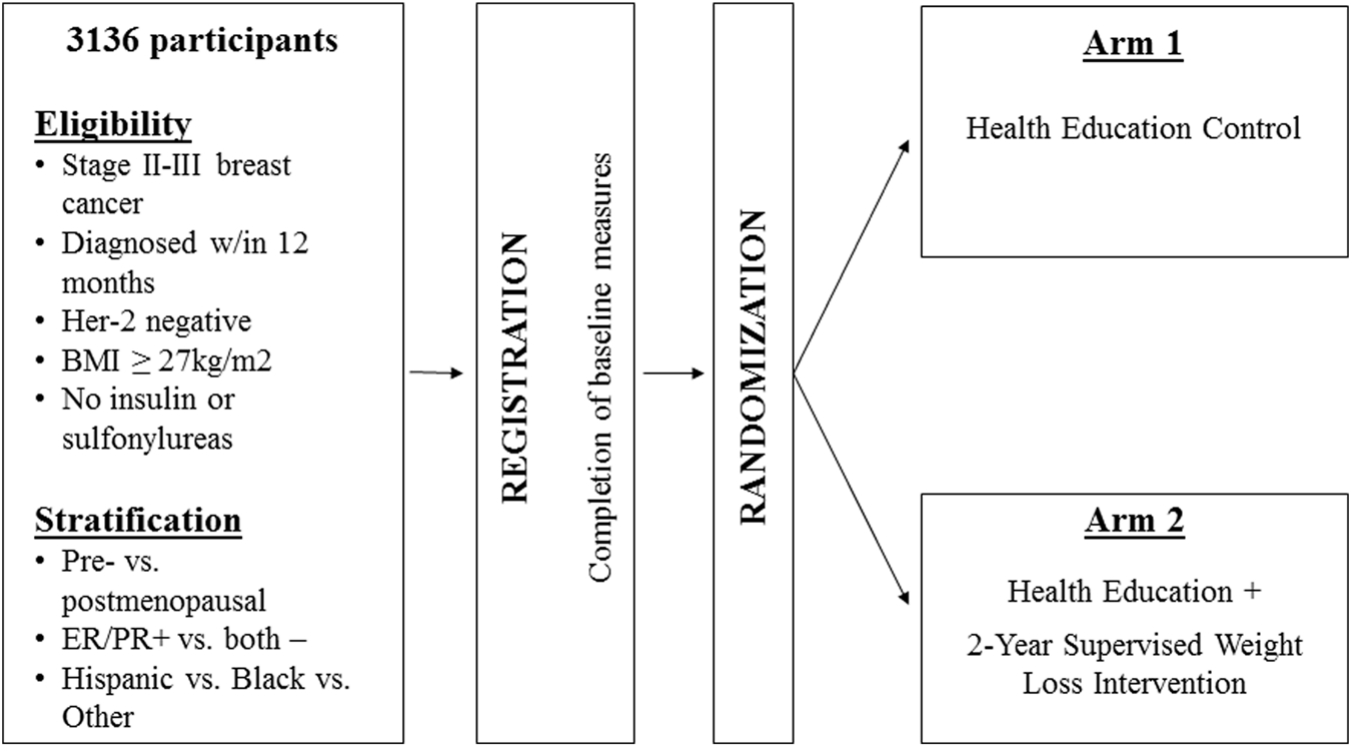
Study Schema

BWEL is a randomized trial evaluating the effect of a weight loss intervention plus health education materials vs. health education materials alone on IDFS in women with early breast cancer (Fig. 1).

Target enrollment is 3136. The trial sponsor is the Alliance, with intergroup participation from NCTN, U.S. NCI Community Oncology Research Program (NCORP) and Canadian Clinical Trials Group (CCTG) sites. Participants are randomized 1:1 to a 2-year telephone-based weight loss intervention plus a health education program or to a health education program alone control group. Patients are enrolled through local clinic sites, and the weight loss intervention is administered centrally from a call center based at the Dana-Farber Cancer Institute. The trial aims for robust minority enrollment, with accrual strategies including the availability of intervention materials in Spanish, outreach efforts to advocacy groups, and partnerships with clinic sites serving minority populations.

### Eligibility

Key eligibility criteria include histologic diagnosis of stage II–III, HER-2 negative breast cancer within the past 12 months. Patients with triple negative breast cancer may have any stage II–III non-inflammatory breast cancer; those with estrogen and/or progesterone receptor positive breast cancer must have involved lymph nodes or a T3 tumor. Participants must be females ≥18 years of age, have a BMI of at least 27 kg/m^2^, be able to read and speak English (the study will open to Spanish speaking patients in 2017), and have completed all chemotherapy, radiation and surgery at least 21 days prior to study enrollment. Participants with metastatic cancer or diabetes treated with insulin or sulfonylurea drugs are ineligible for enrollment. Each participant must sign an IRB-approved, protocol-specific informed consent in accordance with federal and institutional guidelines.

## Study interventions

### Health education program

All patients in both the intervention and control groups receive information focused on breast cancer survivorship. The program includes: yearly mailings of educational materials, such as the National Cancer Institute’s Facing Forward booklet; biannual study newsletters; invitations to webinars regarding breast cancer survivorship; and annual holiday and anniversary cards. All English-speaking participants receive a 2-year subscription to the Harvard Women’s Health Watch, a newsletter focused on topics related to women’s health. Spanish-speaking patients will receive NIH Medlineplus Salud.

### Weight loss intervention

Participants randomized to the weight loss intervention arm receive a 2-year, telephone-based, lifestyle intervention that promotes weight loss through caloric restriction and increased physical activity. The intervention is based on Social Cognitive Theory and adapted from the evidence-based Diabetes Prevention Program (DPP),^19^ Look Ahead,^20^ and Lifestyle Intervention Adjuvant (LISA) studies,^21^ with materials updated to reflect recent research in weight loss and breast cancer.

Intervention participants are assigned a trained coach at the BWEL call center at Dana-Farber Cancer Institute and are provided 42 phone calls over the 2-year intervention period. Calls are delivered weekly for the first 12 weeks, bi-weekly for the remainder of the 1st year, and monthly in the 2nd year. Participants receive a lifestyle workbook to accompany the coaching sessions and have access to a study website to support self-monitoring of diet and physical activity throughout the study. Participants receive a number of tools to assist in meeting weight loss goals, including a wireless scale and wearable activity sensor (Fitbit, Inc.), a food scale (Ozeri Corp), meal replacement shakes (Nestlé Health Science), measuring cups, and the American Cancer Society Healthy Living cookbook. A toolbox approach is used to tailor the intervention to optimize behavior change for individual participants. Examples of toolbox items include alternative dietary plans and coupons for exercise classes or equipment.

### Intervention goals

- Weight loss: 10% of baseline weight (0.5–1.0 kg per week) to a BMI no less than 21 kg/m^2^.
- Caloric intake: calorie deficit of 500 to 1000 kcal/day based on current weight, with levels ranging from 1200–1499 kcal/day for patients weighing less than 250 lbs at baseline and 1500–1800 kcal/day for patients weighing more than 250 lbs.
- Physical activity: gradual increase in moderate intensity aerobic physical activity, with target goal of 150 min/week during initial phase of intervention and 225 - 300 min/week in maintenance phase of intervention.

### Intervention standardization

Delivery of the telephone intervention is standardized using approaches developed as part of the DPP^19^ and the LISA^21^ study. Standardization is applied to training, certification and supervision of coaches; use of semi-scripted telephone calls; recording and centralized review of calls; centralized calculation of weight, activity and calorie goals; and computerized monitoring of weight, diet and activity changes.

## Study measures

The BWEL study was designed to allow participating sites to collect data from study participants using methods common to other NCTN/NCORP group trials, while measures specific to the lifestyle intervention are collected centrally by the BWEL call center and intervention team. Participants undergo study visits at their local site at the time of enrollment (baseline), every 6 months for 3 years, and then annually for an additional 7 years. Study measures collected at sites include anthropometric measures, blood draws, questionnaires, and assessments of disease status and adverse events (Table 2). In addition, a subset of participants complete 24-h dietary recalls and 7-Day physical activity recall interviews, conducted by the Behavioral Measurements and Interventions Share Resource at the University of Arizona Cancer Center, and wear accelerometers to provide physical activity and dietary data (see below).

**Table 2:** BWEL (A011401) study measures and time points

	Baseline	Month 6	Month 12 and 18	Month 24	Month 30 and 36	Annual follow-up
Site collected measures						
History and physical	X	X	X	X	X	X
Height	X					
Weight	X	X	X	X	X	X
Waist and hip circumference	X	X	X	X	X	X
Co-morbidity assessment	X	X	X	X	X	X
Adverse event assessment		X	X	X	X	
Outcome assessment		X	X	X	X	X
Bilateral mammogram	X	(performed yearly while on study as applicable)				
Laboratory						
Fasting glucose (performed at site)	X	X		X		
Fasting serum and plasma (collected and sent to biobank)	X	X		X		
Tissue-based and genomic biomarkers A011401-ST1: for patients who consent to participate						
Fasting whole blood	X	X		X		
Sample of tumor and benign (if available) breast tissue from primary surgery	X					
Centrally collected measures: health behaviors and PRO (A011401-HO1)						
(First 514 patients who enroll on A011401)						
24-h dietary recall, 7-day physical activity recall and accelerometer	X	X		X	X	
(all administered centrally)						
PRO questionnaire booklet	X	X		X	X	

## Correlative studies

### Health behaviors and PRO

Detailed measures of physical activity, dietary intake, and PRO are being collected in a pre-planned, representative subset of 514 intervention and control participants at baseline and at 6, 24 and 36 months. This health behaviors and quality of life correlative study (A011401-HO1) uses a prospective, longitudinal design to assess differences in changes in health behaviors and patient-reported outcomes (PRO) over time between arms (Table 3). Objectives include evaluating the effect of the intervention on quality of life and other PROs, as well identifying predictors of intervention adherence.

**Table 3:** Health behaviors and patient reported outcomes measures

Outcome variable	Study measure
Health behaviors	
Physical activity	Actigraph accelerometer
	7-day physical activity recall interview
Dietary intake	24-h dietary recall interview
Patient-reported outcomes	
Physical function	PROMIS Physical Function (part of PROMIS-29 Profile v2.0)
Quality of life and mood	PROMIS Global Health
	PROMIS 29 Profile v2.0 (anxiety, depression, fatigue, sleep, social roles, pain)
Body Image	Body Image and Clothing (CARES subscales)
Cancer-treatment and menopausal symptoms	PROMIS Applied Cognition-Abilities-Short Form 6a, BCPT, EORTC QLQ-CIPN20 (#1-6), Global Ratings of Change in Joint and Stiffness

*PROMIS* patient-reported outcomes measurement information system, *BCPT* breast cancer prevention trial, *EORTC QLQ-CIPN* European Organization for Research and Treatment of Cancer, quality of life questionnaire-chemotherapy-induced peripheral neuropathy

### Correlative science

Correlative analyses will allow for the exploration of intermediate biomarkers meditating the relationship between obesity and breast cancer. Fasting plasma and serum are collected from all study participants at baseline and at 6 and 24 months. Patients who consent to an optional sub-study (A011401-ST1) also provide fasting whole blood samples and a block of tumor and benign breast tissue collected at the time of breast surgery (A011401-ST1). Planned correlative analyses are described in Table 4. A central biobank of blood, tumor tissue and benign breast tissue has been established and a correlative science subcommittee will review scientific advances as the trial progresses, add assays supported by emerging evidence, and review proposals for additional analyses with stored blood and tumor tissue.

**Table 4:** Correlative science

Specimen type	Planned analyses
Blood	
Fasting serum/plasma	Insulin, glucose, adipocytokines, inflammatory mediators
Fasting whole blood	DNA methylation, telomere length and activity
Tissue	
Tumor	Gene expression, immunohistochemical and other markers related to potential obesity effects such as insulin and leptin receptor and pathway activation (e.g., PI3K, JAK-STAT)
Benign breast tissue	Examined for evidence of localized inflammation, including crown-like structures

*DNA* deoxyribonucleic acid

## Statistical considerations

This randomized controlled trial of a weight loss intervention in overweight and obese women with early stage breast cancer consists of two arms: a telephone-based intervention group and a health education control group. Patients are randomized 1:1 within stratification factors: menopausal status (premenopausal vs. postmenopausal), hormone receptor status of the tumor (ER and/or PR positive vs. ER and PR negative), and race/ethnicity (African American vs. Hispanic vs. Other).

The primary objective of this trial is to compare the effect of the telephone-based intervention vs. a health educational control on IDFS. The total sample size is 3136 patients. Patient follow-up for primary and secondary endpoints will continue to a maximum of 10 years from trial enrollment.

### Sample size, accrual time and study duration

The primary comparison of IDFS among intervention assignments will be performed in the intent-to-treat (ITT) population of all randomized patients using a stratified logrank test with a one-sided type I error rate of 0.025.^22^ As a secondary analysis to estimate the effects of the intervention, we will use a multivariate Cox proportional hazard model to evaluate differences between arms after adjusting for stratification factors and the additional covariates of known importance to breast cancer including age, tumor size, number of involved nodes, and tumor grade.^22^ The 5-year IDFS in the control educational arm is assumed to be 77%, based on results from the control arm of E5103^23^ where patients with stage II and III breast cancer received standard adjuvant chemotherapy.

A target hazard ratio of 0.80 between groups would be clinically meaningful and equates to a 4.1% absolute improvement in IDFS in the intervention arm over the assumed rate in the control arm. Final analysis will occur after 739 events of invasive disease are observed, to provide 85% power to reject the null hypothesis under the target hazard ratio. Accounting for an accrual period of 4 years, 4 years of additional follow-up, interim analysis plans for IDFS, and a constant hazard of loss-to-follow-up in the ITT population of 5% by 4 years due to withdrawn consent or other reasons, requires a total sample size of 3136 patients.

### Study monitoring and interim analysis

Statistical analyses will be conducted by the Alliance Statistics and Data Center. Interim monitoring will be conducted semi-annually by the Alliance Data and Safety Monitoring Board (DSMB). Under standard monitoring procedures, the DSMB will monitor study accrual and adverse events. The DSMB will also consider the evidence regarding safety, i.e., adverse events and the feasibility of completing the trial. In addition, two formal interim analyses are planned: (1) to determine whether the effectiveness of the intervention induces sufficient weight change, as measured by change in BMI after 12 months, and (2) to evaluate clinical benefit in terms of IDFS in a group sequential design.

#### Interim analysis for weight change

An analysis for futility to achieve weight loss will be conducted when 25% of the study population has reached the 12-month evaluation point. The DSMB will be provided descriptive statistics for BMI-change by arm. A 4% between group difference in mean percent weight change from baseline to 12-months will be used to define the threshold for minimum effectiveness of the intervention.

#### Interim analysis for primary endpoint

Early stopping for superiority and futility is defined under a one-sided hypothesis of clinical benefit and will be conducted using standard group sequential designs for phase III studies of time-to-event outcomes. Specifically, the *Z*-statistics from stratified long-rank tests will be calculated at each interim analysis, and compared to stopping boundaries using the Lan-DeMets error-spending function (1983). Interim monitoring will begin after 50% of the target IDFS events are reached and continue on a semi-annual basis.

### Trial status

The BWEL study was activated on August 29, 2016. The study is currently open to accrual at more than 1000 sites in the United States. The study was also approved by the CCTG and is anticipated to open in Canadian centers in the Fall of 2017. Accrual as of July 15, 2017 is 517 participants; accrual is anticipated to be completed in 2020, with results available in approximately 2023.

### Data availability

The data are not yet available for the BWEL study as the trial is still in progress.

ClinicalTrials.gov Identifier: NCT02750826
